# A new era for *Italian Journal of Pediatrics*

**DOI:** 10.1186/1824-7288-34-1

**Published:** 2008-11-18

**Authors:** Sergio Bernasconi

**Affiliations:** 1 Department of Pediatrics, University of Parma, Parma, Italy

## Abstract

On behalf of the Editorial Board, welcome to the new *Italian Journal of Pediatrics*, the official journal of the ISP/SIP (Italian Society of Pediatrics/Società Italiana di Pediatria), now publishing on BioMed Central's open access publishing platform. The move to BioMed Central will benefit authors by having their manuscripts published faster with rapid global dissemination. Readers will also benefit from free online access to the journal via the website and a range of full text archives.

## Editorial

The journal was founded more than 30 years ago and has been the leading Italian journal of pediatrics for many years, playing a pivotal role in training and updating Italian pediatricians. In 2001 the Society decided to broaden the international visibility of the journal by publishing the journal in English and accepting articles from authors in other countries. Following this, *Italian Journal of Pediatrics *was included in PubMed Central in 2005. These successful results were obtained thanks to the hard work of several Editors-in-Chief and Editorial Boards, and, in particular, the most recent Editor-in-Chief, Prof. Mario De Curtis. Last year, the ISP/SIP decided to go a step further, and to publish the journal only online. I was honored to be nominated Editor-in-Chief, and to have the opportunity to work with an international Editorial Board of a high scientific level.

The journal will continue to publish new research in all areas of pediatrics, particularly focusing on assisting young investigators in the field, establishing debate and paying special attention towards pediatrics in developing countries. Topics of particular interest for *Italian Journal of Pediatrics *include:

- practical application of basic research

- quality of care in children

- national and international guidelines following evidence based medicine

- clinical and scientific training

Over the next few years we shall work to build up a journal with a successful international reputation, publishing high quality research on all areas of pediatrics. In order to obtain these results, we look forward to receiving your high quality submissions, general feedback and suggestions or criticisms for improving the journal.

